# METTL3 regulates hippocampal gene transcription via N6-methyladenosine methylation in sevoflurane-induced postoperative cognitive dysfunction mouse

**DOI:** 10.18632/aging.203604

**Published:** 2021-10-05

**Authors:** Baiqing He, Jian Wang

**Affiliations:** 1 Department of Anesthesiology, The Affiliated Hospital of XiangNan University, Chenzhou, Hunan, China; 2 Department of Anesthesiology, Shaoxing Yuecheng People’s Hospital, Shaoxing, Zhejiang, China

**Keywords:** POCD, m6A methylation, METTL3, sevoflurane, hippocampus

## Abstract

Elderly patients are prone to cognitive impairment and memory loss after surgical operations. This perioperative cerebral damage, named postoperative cognitive dysfunction (POCD), is profoundly affected by anesthesia. N6-methyladenosine (m6A) RNA methylation is a widely-studied epigenetic modification to regulate gene expression; however, is has never been studied in POCD. In the present study, elderly POCD mouse models were constructed using sevoflurane, and we observed a compromised global m6A RNA methylation in the mice’s hippocampuses compared with the control. Our RIP-Seq data suggested that 1244 genes (SOX2, SYN1, and BDNF) showed m6A RNA methylation in their 5′UTRs, which was significantly lower than that in the control; while only 56 genes (BACE1 and IL17A) showed m6A RNA methylation in their 5′UTRs, which was significantly higher than that in the control. Unexpectedly, m6A RNA methylation with significant differences in exons, introns, or 3′UTRs was observed in only few genes. Although we failed to find any differences in the expression of m6A-associated proteins, such as m6A “writers”, “erasers”, and “readers”, between the sevoflurane treatment and control groups, RIP-qPCR assays indicated that the binding affinity of METTL3 on mRNA 5′UTRs was particularly weakened in target genes by sevoflurane. Finally, we found that phosphorylation of METTL3 could be reduced by sevoflurane because of the inactivation of the MAPK/ERK pathway. Overall, our study determined that the inactivation of METTL3 in the mouse hippocampus, induced by sevoflurane-mediated MAPK/ERK suppression *in vivo*, resulted in a perturbation in m6A RNA methylation signals in the pathogenesis of POCD.

## INTRODUCTION

Postoperative cognitive dysfunction (POCD) is a common result of perioperative cerebral damage in elderly patients, and its symptoms include cognitive impairment, memory loss, learning disabilities, information processing disorders, and delirium [1]. POCD is still difficult to predict and evaluate owing to the lack of formal diagnostic criteria as well as the subtle nature of the cognitive changes [2]. Although cognitive change after anesthesia and surgery was described over 100 years ago—initially as delirium and dementia—and POCD has gained attention from scholars in recent years [3], existing studies have been plagued with experimental problems, such as variable test batteries, a lack of control groups, the loss of patients during follow-ups, and inconsistent intervals between testing periods [4]. Thus, to date, inconclusive research and POCD’s confusing pathogenesis have together mand developing treatments, rehabilitative medicine, and accurate diagnosis criteria for POCD difficult.

Researchers have come up with various hypotheses to reveal the mechanisms of POCD. One of the top potential risk factors for developing POCD is the impact of general anesthesia [5]. Frequently used narcotics can be classified into inhalational and intravenous anesthesia. The general inhaled anesthetics such as sevoflurane can increase the risk of Alzheimer's disease (AD) in the aging brain [6] and exert neurotoxic effects through neuroinflammation [7], caspase-mediated apoptosis [8], mitochondrial dysfunction, and oxidative stress [9]. Recently, sevoflurane has been utilized to establish a POCD rodent model to explore the underlying mechanism of POCD’s pathogenesis [10, 11], indicating that sevoflurane is implicated in the causation of the neurological and cognitive deficits seen in POCD.

N6-methyladenosine (m6A) RNA methylation is a type of abundant and reversible RNA modification in eukaryotes that can control mRNA maturation, alternative splicing, localization, structural folding, and protein translation [12]. M6A RNA methylation is installed by the m6A methyltransferases (METTL3/14, WTAP, RBM15/15B, and KIAA1429, termed as “writers”), reverted by the demethylases (FTO and ALKBH5, termed as “erasers”), and recognized by m6A binding proteins (YTHDF1/2/3, IGF2BP1, and HNRNPA2B1, termed as “readers”) [13]. Accumulating evidence has shown that m6A RNA methylation plays a crucial and extensive role in RNA production/metabolism and participates in the pathogenesis of multiple diseases. However, m6A RNA methylation has never been implicated in POCD.

In our study, we constructed a POCD mouse model by sevoflurane and performed RIP-Seq assays to study the dynamic changes in global m6A RNA methylation in POCD. We focused on the m6A RNA methylation in different regions of mRNA and further unmask the dysfunction of the key enzyme METTL3, which contributes to m6A RNA methylation abnormalities. Our data will be beneficial for advancing our understanding of the molecular mechanism on the pathogenesis of POCD and will provide potential diagnostic predictors as well as therapeutic targets for POCD.

## RESULTS

### The reduced total m6A RNA methylation in a POCD hippocampus

First, physiological parameters including blood glucose, SpO2, and IL-1β levels for the exclusion of probable diseases of respiratory depression, infection, or hypoglycemia were screened in the mice enrolled in this study (Table 1). Thirty mice were treated with 2% sevoflurane to establish a POCD model. Of these mice, seven were identified as having POCD based on the data from Morris water maze tests conducted before and after the sevoflurane treatments. Another twenty mice receiving normal air were considered as negative controls. Given the escaping latent period, the total distance of movement, and number of grids crossed, the POCD mice took a significantly longer time to seek their destination compared with the controls and non-POCD mice, indicating that POCD mice might have sustained a certain extent of substantial hippocampal memory damage (Table 1).

**Table 1:** The principle indexes of open field test of POCD mice in this study.

**Group**	**Sample size**	**Weight (g)**	**Blood glucose (mmol/L)**	**SpO_2_ (%)**	**IL-1β (pg/m)**	**Average distance (cm)**	**Total across grids**	**Escaping latent period (s)**
Control	20	24.1 ± 6.2	8.8 ± 2.1	97.44 ± 1.36	8.34 ± 1.56	36.25 ± 4.8	76.3 ± 9.8	48.9 ± 5.6
Non-POCD	23	23.6 ± 9.2	8.5 ± 2.3	96.67 ± 1.09	7.66 ± 2.36	32.34 ± 5.9	77.6 ± 12.5	54.7 ± 7.3
POCD	7	24.8 ± 3.1	9.1 ± 3.0	96.37 ± 1.37	8.14 ± 2.25	18.77 ± 6.2^*#^	39.4 ± 8.6^*#^	90.6 ± 11.8^*#^

^*^means the statistical significance vs. control with *p* < 0.05

^#^means the statistical significance vs. non-POCD with *p* < 0.05

**Figure 1:**
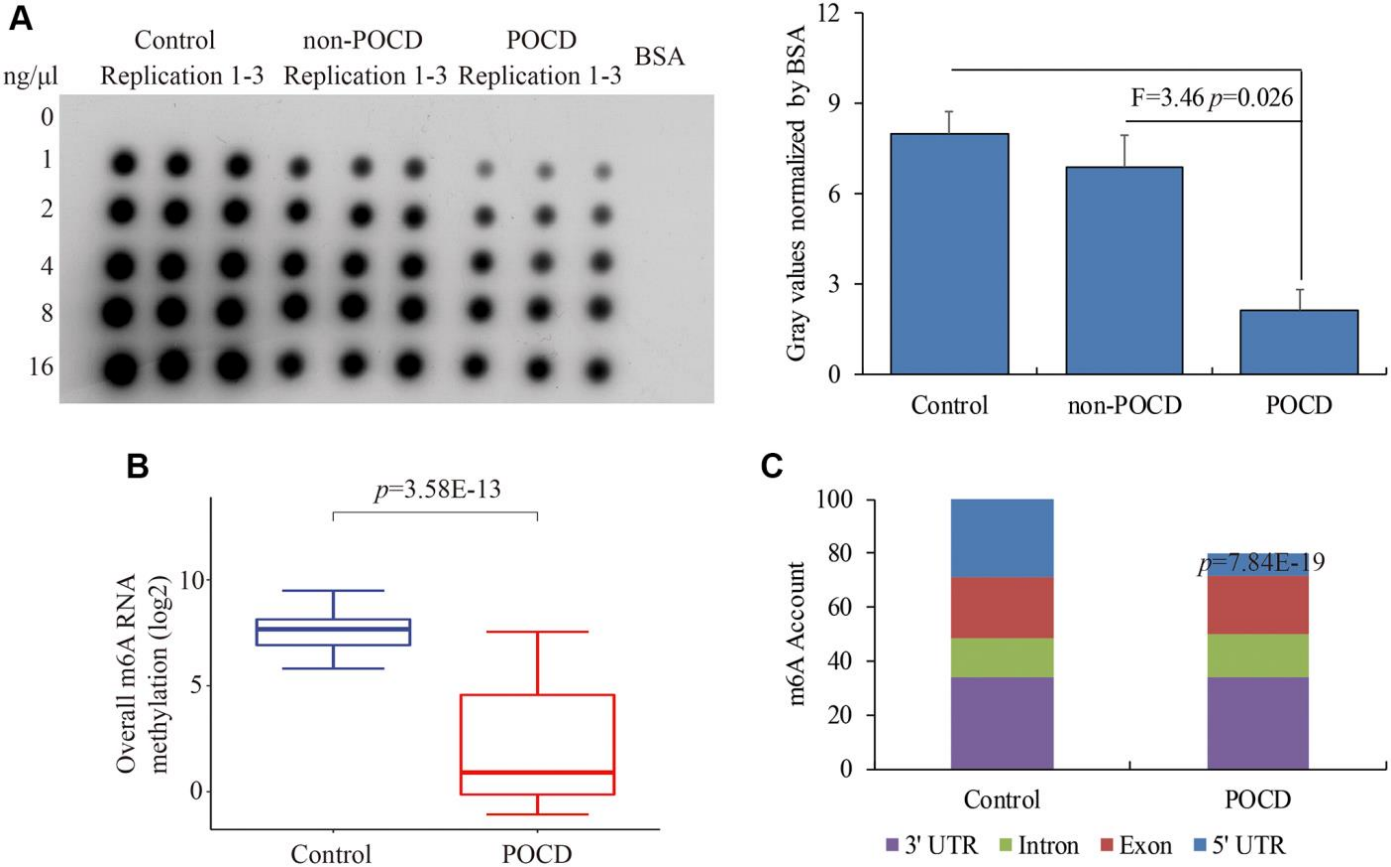
**The global m6A RNA methylation in the hippocampus of POCD mice.** The m6A RNA methylation levels detected by dot plot assay (**A**) and RIP-seq (**B**). The distribution of differential m6A enrichment on the entire transcriptome (**C**). Data are presented as the mean ± standard error of the mean of three individual experiments; *p* < 0.05 vs. control or non-POCD groups.

Total hippocampal RNA was used to further detect m6A RNA methylation levels by dot plot assays. We observed a compromised global m6A RNA methylation pattern in POCD compared with the control (*F* = 5.82, *p* = 0.017) and non-POCD mice (*F* = 3.46, *p* = 0.026) (Figure 1A). Next, RIP-Seq was conducted to investigate the dynamics of m6A RNA methylation to compare the hippocampuses of POCD mice with those of the controls. The clean reads and unique mapping ratio both indicated a high quality of sequencing data (Table 2). Consistently, the genome-scale m6A RNA methylation was significantly lower in the POCD mice than in the controls (*p* = 3.58E-13) (Figure 1B), which was mainly attributed to the compromised m6A RNA methylation especially at the 5′UTR (*p* = 7.84E-19) (Figure 1C). Taken together, our data indicated a reduced global m6A methylation in the POCD hippocampus.

**Table 2:** The basic information of RIP-sequencing data.

**Sample**	**Control_Input_rep1**	**Control_Input_rep2**	**Control_m6AIP_rep1**	**Control_m6AIP_rep2**	**Treated_Input_rep1**	**Treated_Input_rep2**	**Treated_m6AIP_rep1**	**Treated_m6AIP_rep2**
Total Raw Reads	24621956	20850293	22948365	26199989	24783948	24085547	25259459	23060216
Total Raw Bases	1.85E + 09	1.56E + 09	1.72E + 09	1.96E + 09	1.86E + 09	1.81E + 09	1.89E + 09	1.73E + 09
Total Clean Reads	24176175	20470451	22463594	25646939	24289133	23604466	24749131	22594158
Total Clean Bases	1.74E + 09	1.47E + 09	1.6E + 09	1.83E + 09	1.74E + 09	1.69E + 09	1.77E + 09	1.61E + 09
Mapped Reads	22726965	19240240	21089697	24073135	22938393	22291299	23193605	21173862
Mapped Ratio	94.01%	93.99%	93.88%	93.86%	94.44%	94.44%	93.71%	93.71%
Uniquely Mapped Reads	19775728	16734484	19953213	22774629	19958368	19388196	21821048	19916679
Uniquely Mapped Ratio	81.80%	81.75%	88.82%	88.80%	82.17%	82.14%	88.17%	88.15%

### The differential m6A RNA methylation especially on the 5′UTR in POCD

**Figure 2:**
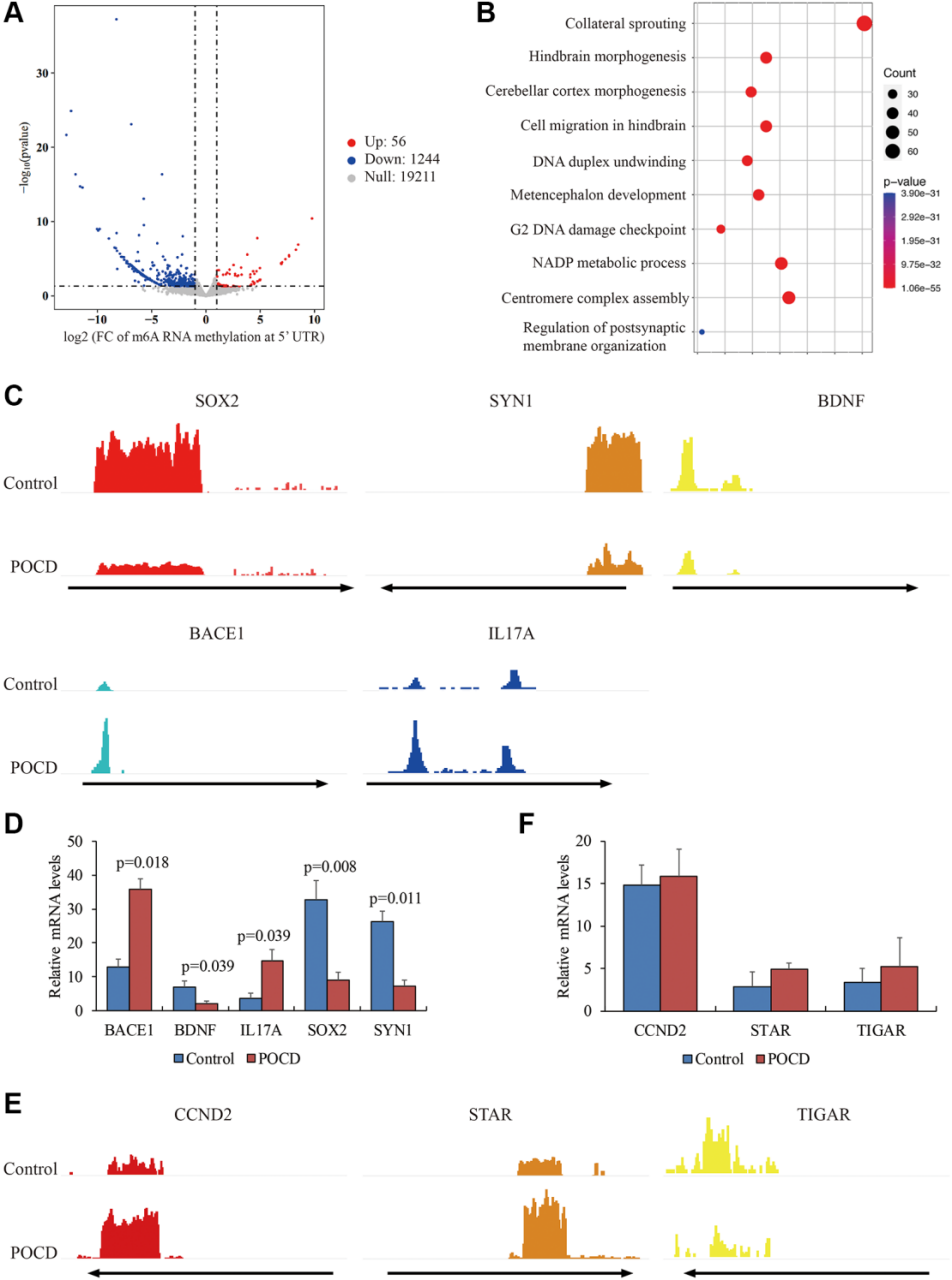
**Differential m6A RNA methylation at the 5′UTR in POCD.** The genes with differential m6A RNA methylation at the 5′UTR in POCD mice depicted by a volcano plot. (**A**) The GO analysis of these genes is depicted by a bubble chart. (**B**) The m6A enrichment at the 5′UTR of *SOX2*, *SYN1*, *BDNF*, *BACE1,* and *IL17A* in POCD mice depicted by IGV. (**C**) The mRNA levels of *SOX2*, *SYN1*, *BDNF*, *BACE1*, and *IL17A* in POCD. (**D**) The m6A enrichment at the 3′UTR of *CCND2*, *STAR*, and *TIGAR* in POCD shown by IGV. (**E**) The mRNA levels of *CCND2*, *STAR,* and *TIGAR* in POCD. (**F**) Data are presented as the mean ± standard error of the mean of three individual experiments; *p* < 0.05 vs. control group.

Next, we observed that m6A RNA methylation was significantly reduced in 1,244 genes and elevated only in 56 genes in POCD mice compared with the controls (Figure 2A). These genes were involved in central nervous system development and the DNA damage response, NADP (Figure 2B), containing *BACE1*, *BDNF*, *IL17A*, *SOX2*, and *SYN1* (Figure 2C). Transcription of these genes was also validated by qPCR. It was noteworthy that the genes with robustly enriched m6A RNA methylation at the 5′UTR were all highly expressed, while genes with infrequent m6A RNA methylation at the 5′UTR were transcriptionally repressed (Figure 2D). Nevertheless, we failed to find any significant differences in the mRNA levels of *CCND2*, *STAR*, and *TIGAR*, which had differential m6A RNA methylation at the 3′UTR (Figure 2E and 2F). Taken together, we determined that abnormal m6A RNA methylation at the 5′UTR might contribute to transcription profiling in POCD.

### The weakened METTL3 affinity for target genes in POCD

**Figure 3:**
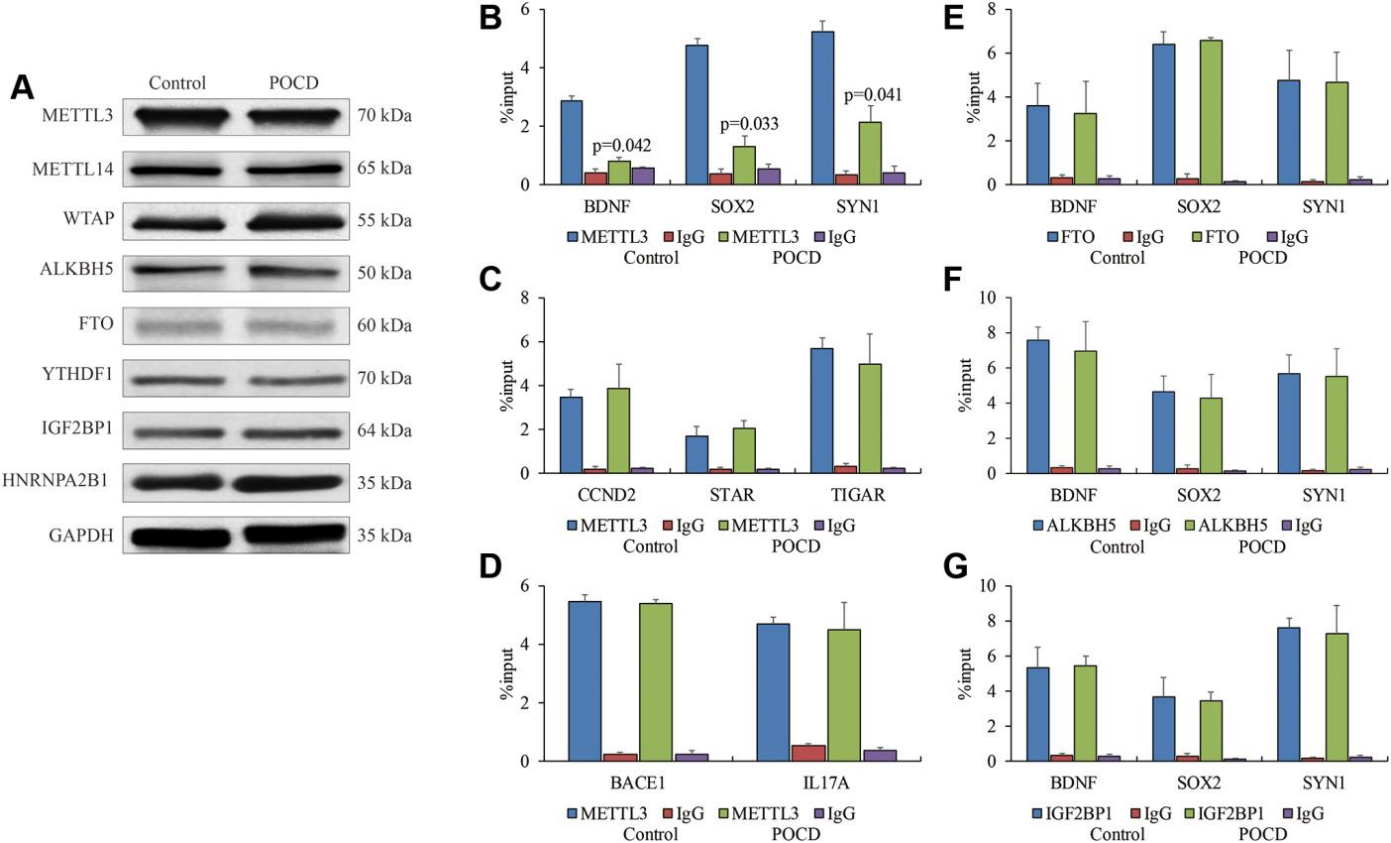
**The weakened binding affinity of METTL3 in POCD.** The protein levels of METTL3, METTL14, WTAP, ALKBH5, FTO, YTHDF1, IGF2BP1 and HNRNPA2B1 in the hippocampuses of POCD mice. (**A**) The binding affinity of METTL3 on *BDNF*, *SOX2*, and *SYN1*, (**B**) *CCND2,* *STAR*, and *TIGAR*, (**C**), as well as *BACE1* and *IL17A* (**D**) in hippocampal neurons of POCD mice shown by RIP-qPCR. The binding affinity of FTO, (**E**) ALKBH5 (**F**) and IGF2BP1 (**G**) on *BDNF*, *SOX2*, and *SYN1* in hippocampal neurons of POCD mice shown by RIP-qPCR. Data are presented as the mean ± standard error of the mean of three individual experiments; *p* < 0.05 vs. control group.

Next, we investigated the role of enzymes regulating m6A RNA methylation at the 5′UTR in POCD. Expressions of *METTL3*, *METTL14*, *WTAP*, *FTO*, *ALKBH5*, *YTHDF1*, *IGF2BP1*, and *HNRNPA2B1* showed no significant differences between the POCD mice and the controls (Figure 3A). However, the affinity of METTL3 for target genes *BDNF*, *SOX2,* and *SYN1* with reduced m6A RNA methylation at the 5′UTR was compromised in the POCD mice compared with the controls (Figure 3B). In turn, it was strange that enrichments of METTL3 on genes *BACE1* and *IL17A* with elevated m6A RNA methylation at the 5′UTR in the POCD mice did not change compared with the controls (Figure 3C). Additionally, the interaction of METTL3 on genes *CCND2*, *STAR*, and *TIGAR* with the 3′UTR did not change in the POCD mice compared with the controls (Figure 3D). We also examined the binding ability of the three enzymes FTO, ALKBH5, and IGF2BP1 for regulating m6A RNA methylation on *BDNF*, *SOX2*, and *SYN1* by RIP-qPCR, and we failed to find any differences in the 5′UTR (Figure 3E–3G). Collectively, the weakened METTL3 affinity was likely to contribute to the reduction of m6A RNA methylation at the 5′UTR of RNAs in POCD.

### METTL3 function suppressed by sevoflurane-induced inactivated MAPK/ERK

**Figure 4:**
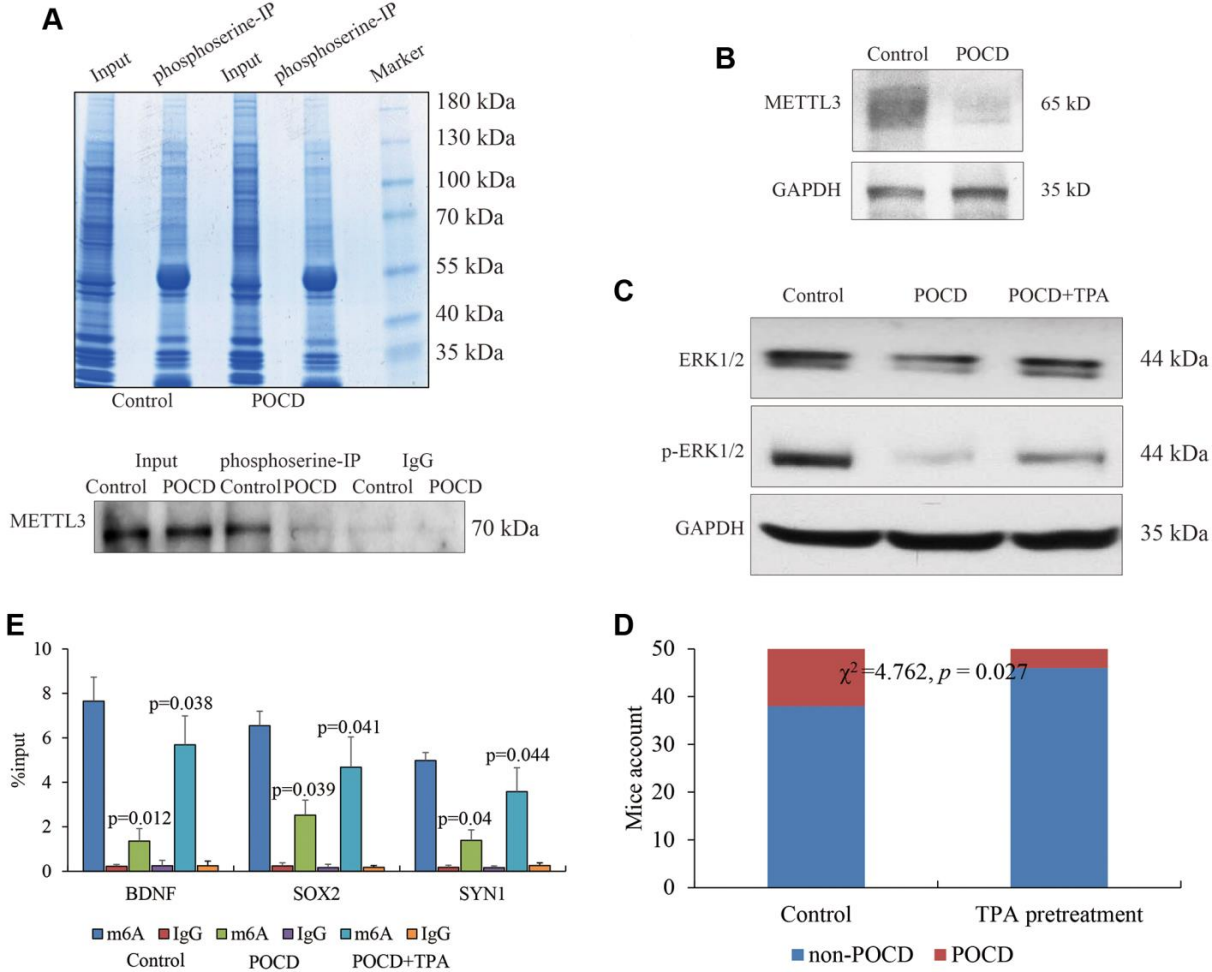
**METTL3 activity affected by the MAPK/ERK pathway.** Phosphorylated METTL3 pulled down by phosphoserine. (**A**) METTL3 expression in POCD (**B**) and the activity of the MAPK/ERK pathway in the hippocampal neurons of mice with sevoflurane-induced POCD and TPA treatment (**C**) shown by regular WB. The m6A RNA methylation at the 5′UTR of *BDNF*, *SOX2*, and *SYN1* in POCD mice and additional TPA treatment. (**D**) POCD occurrence comparison between sevoflurane alone and TPA pretreatment by Chi-square analysis. (**E**) Data are presented as the mean ± standard error of the mean of three individual experiments; *p* < 0.05 vs. control group.

Finally, we attempted to bridge the inactivated METTL3 and sevoflurane-induced POCD. Previous studies revealed that the serine phosphorylation of METTL3 played a crucial role in METTL3’s activity and was dependent on the MAPK/ERK signaling pathway [14]. To this end, IP experiments were conducted to capture phosphoserine, and reduced phosphorylated METTL3 was observed in the POCD models’ hippocampuses (Figure 4A). Due to the lack of a commercial phosphorylated METTL3 antibody, we indirectly found less bands of METTL3 over 65 kD in the POCD models’ hippocampuses using a regular METTL3 antibody by WB (Figure 4B), which indicated a potential reduced protein modification affected by sevoflurane. Moreover, the ERK activator TPA was used to treat the other three POCD mice by intracerebroventricular injection. The activity of the MAPK/ERK signaling pathway in hippocampal neurons was abrogated in the POCD mice compared with the controls and rescued by TPA (Figure 4C), suggesting that sevoflurane affected METTL3’s activity through MAPK/ERK inhibition. Expectedly, the target genes *BDNF*, *SOX2*, and *SYN1* were observed to have highly enriched m6A RNA methylation at their 5′UTR after TPA treatment compared with the POCD mice (Figure 4D).

Our data supported the conclusion that sevoflurane inactivated the MAPK/ERK pathway, which caused dysfunction of METTL3. Fifty elderly mice were pretreated with the ERK activator TPA followed by treatment with sevoflurane to induce POCD. Compared with the fifty mice that were only treated with sevoflurane, the occurrence of POCD in the fifty mice pretreated with TPA was significantly decreased (χ^2^ = 4.762, *p* = 0.027) (Figure 4E). Taken together, we determined that the activation of the MAPK/ERK pathway was beneficial to METTL3’s functional maintenance for m6A RNA methylation against POCD.

## DISCUSSION

Although the roles of m6A RNA methylation have garnered increasing attention in multiple human diseases, they have yet to be investigated in POCD. M6A RNA methylation is abnormally high in AD, which is attributed to elevated METTL3 levels and compromised FTO [15]. Conversely, other studies have declared that the hippocampal METTL3 is beneficial to long-term memory maintenance and consolidation in mice [16] and is decreased in AD [17], indicating that METTL3 may enhance the translation of neuronal early response genes via m6A RNA methylation. Based on the current data, we confirmed that the global m6A methylation level is reduced in the hippocampus of the sevoflurane-induced POCD mouse model, and m6A RNA methylation governs the expression of genes associated with efficacy of memory and learning as well as neurons’ differentiation and morphogenesis. Nevertheless, as a newly revealed method of epigenetic regulation, m6A RNA methylation seems to be responsible for a small proportion of genes’ expression repression in POCD. Notably, m6A enrichment at the 5′UTR but not at the 3′UTR or other regions indeed plays an important role in modulating gene expression. M6A residues at the 5′UTR enable a cap-independent model of translation initiation by circumventing the m7G cap requirement [18], while the enrichment of m6A in the 3’ terminal of mature polyadenylated mRNAs is associated with an alternative polyadenylation [19]. We speculated that in the system of hippocampal neuron cells, m6A-mediated translational processes occupy a dominant stage for final gene expression.

We examined all the enzymes associated with m6A RNA methylation, and we observed that the binding ability of METTL3 to target genes is compromised in POCD with the inactivation of METTL3. The relationship between posttranslational modifications of METTL3 and METTL3’s function has been comprehensively studied [14]. Multiple potential serine sites on METTL3 for phosphorylation are also documented; however, commercial antibodies for specific phosphorylated METTL3 are unavailable. Therefore, our IP results could only verify that phosphorylated METTL3 is substantially eliminated, but we could not explore which serine sites contributed to METTL3’s activity in POCD. Interestingly, the weakened affinity of METTL3 is limited to the 5′UTR, which can affect gene expression. From these results, simply understanding the activity of METTL3 is not enough to determine the epigenetic mechanism of m6A RNA methylation on gene expression. In this study, we speculated two possibilities for the epigenetic mechanism: (1) Different combinations of phosphorylation at multiple sites on METTL3 may determine the interaction of METTL3 and the target gene, and (2) multiple unknown RNA binding proteins may coordinate to facilitate the recruitment of these enzymes to target genes against POCD. Both possible mechanisms deserve further investigation.

Overall, our study determined that the inactivation of METTL3 in the mouse hippocampus induced by sevoflurane-mediated MAPK/ERK suppression *in vivo* resulted in a perturbation in m6A RNA methylation signals in the pathogenesis of POCD.

## MATERIALS AND METHODS

### Animal study

A POCD mouse model was established as previously described [10, 11]. In brief, fifty 40-week-old outbred female C57BL/6 mice purchased from the experimental animal center of Xiangnan University were enrolled in this study. Peripheral blood isolated from the caudal vein was used to access the glucose and oxygen saturation (SpO2) levels via a biochemical analyzer (Beckman coulter, Brea, USA) as well as IL-1β levels by ELISA kits (R&D systems, Minneapolis, USA). All the mice were involved in the Morris water maze test by EthoVision XT working system (Noldus, Netherlands) as indicated by the manufacturer’s instructions. The escaping latent period of each mouse was recorded. Thirty mice were randomly selected and given 2% sevoflurane for 4 h in an automatic anesthetic chamber with a size of 24 cm × 12 cm × 18 cm (Rurui Technology Co., Ltd, Guangzhou, China). After natural resuscitation, POCD and non-POCD mice were picked based on their escaping latent periods. The other twenty mice received normal air and were treated as negative controls. Mice were pretreated with 5 mM/kg MAPK/ERK activator phorbol 12-myristate 13-acetate (TPA) (N2060, APExBIO, Houston, TX, USA) for 6 h before sevoflurane induction via intracerebroventricular injection. The mice were sacrificed via cervical dislocation, and their hippocampus were harvested immediately, washed with cold PBS, and RIPA buffer or Trizol Reagent were added accordingly. All the procedures were followed by the Institutional Animal Care and Use Committee of XiangNan University (animal protocol number 2017-32-166).

### Dot plot

Dot plot was performed as previously described [20]. In brief, total RNA was isolated by the TRIzol method as described in the subsequent RT-qPCR section of the Materials and Methods. RNA samples dissolved in three times their volumes of RNA incubation buffer were denatured at 65°C within 5 min. Then the samples, divided into subgroups of 400 ng, 200 ng, and 100 ng, were loaded onto an Amersham Hybond-N+ membrane (GE Healthcare, Pittsburgh, PA, USA) installed in a Bio-Dot Apparatus (Bio-Rad, Richmond, CA, USA) with a mixture of ice-cold 20 × SSC buffer (Millipore, Billerica, MA, USA). The membrane was UV cross-linked for 5 min and washed with PBST. Then the membrane was stained with 0.02% methylene blue (Shanghai Sangon Biotechnology Company, Shanghai, China) and scanned to determine the total content of input RNA. After being blocked with 5% nonfat milk, the membrane was incubated with a specific m6A antibody (1:1000, Millipore) overnight at 4°C. Dot blots were hatched with HRP-conjugated anti-mouse immunoglobulin G (IgG) for 1 h before being visualized by an imaging system (Bio-Rad).

### RNA immunoprecipitation (RIP) assay

The genome-wide m6A was detected by an RIP kit (Millipore). Hippocampal neuron cells were washed with pre-cooled PBS, and the supernatant was discarded. RIP Lysis Buffer was added, and the products were lysed on ice and collected. Then, the beads were washed with RIP Wash Buffer. After removing the supernatant, the beads were incubated with 1 μg antibodies of m6A (56593S, CST, Beverly, MA, USA), METTL3 (86132, CST), FTO (31687, CST), ALKBH5 (16837-1-AP, Proteintech, Rosemont, IL, USA), and IGF2BP1 (22803-1-AP, Proteintech) for 30 min at room temperature, and normal rabbit immunoglobulin G (Abcam, Cambridge, MA, USA) was used as the control. Subsequently, the cell lysate was incubated with RIP buffer containing magnetic beads. RNA was extracted after proteinase K digestion and used for subsequent sequencing or RT-qPCR assays.

For sequencing, the concentration and quality of purified RIP RNAs were measured by a Nanodrop 2000 (Thermo Fisher Scientific) and Agilent bioanalyzer 2100 (Agilent, Santa Clara, CA, USA). A total of 4 μg RNA in each group was used for library preparation by NEBNext Ultra Directional RNA Library Prep Kit for Illumina (NEB, Ipswich, MA, USA) following the manufacturer’s instructions and sequenced on an Illumina Hiseq platform.

The raw data, deposited into the ArrayExpress database (https://www.ebi.ac.uk/arrayexpress) with the accession number E-MTAB-10510, were adaptors-trimmed, the low quality reads were filtered out using Trimmomatic (non-default parameters: SLIDINGWINDOW:4:15 LEADING:10 TRAILING:10 MINLEN:35) [21], and the quality of the clean reads was checked using Fastqc [22]. Next, the clean reads were aligned to the latest mouse genome assembly 10 mm using Hisat2 v2.0.5 (non-default parameters: --rna-strandness RF --dta) [23]. The transcripts were assembled, and the expression levels were estimated with FPKM values using the StringTie algorithm (non-default parameters: --rf) [24]. Differential mRNA and lncRNA expression among the groups were evaluated using an R package Ballgown [25], and the significance of differences by the Benjamini and Hochberg (BH) *p*-value adjustment method was computed. Gene annotation was described by the Ensembl genome browser database (http://www.ensembl.org/index.html). The R package ClusterProfiler was used to annotate the differential genes with gene ontology (GO) terms and the Kyoto Encyclopedia of Genes and Genomes (KEGG) pathways [26]. The peaks of m6A of certain genes were browsed with an Integrative Genomics Viewer (IGV).

### Reverse transcription quantitative PCR (RT-qPCR)

Total RNA was extracted from tissues and cells using TRIzol Reagent (Invitrogen). The total RNA and RNA pulled down from RIP were reversely transcribed into complementary DNA (cDNA) in accordance with the instructions of the Primescript™ RT Reagent Kit (TaKaRa Biotechnology, Dalian, China) and One Step PrimeScript miRNA cDNA Synthesis Kit (TaKaRa Biotechnology), respectively. RT-qPCR was performed on an ABI7500 Quantitative PCR instrument (Applied Biosystems, Foster City, CA, USA) using SYBR^®^ Premix Ex Taq™ Kit (TaKaRa Biotechnology) under the following conditions: 95°C for 2 min followed by 50 cycles at 95°C for 5 s, 60°C for 10 s, 72°C for 30 s, and 72°C for 10 min. The relative expression of products was calculated by the 2^−ΔΔCt^ method. The experiment was repeated three times. All primers synthesized by Shanghai Sangon Biotechnology Company are listed in Table 3.

**Table 3:** All primers used in this study.

	**Official symbol name**	**Primer sequences**
For mRNA detection	BACE1	AGACCCACATTCCCAACATCTTTTCC
		CGGGAACTTCTCCGTCGAGGAGGC
	BDNF	GGGTTCGGCGCCACTCCGACCCTGCC
		GGCCCGAACATACGATTGGGTAGTTCGGC
	CCND2	TGATTGCAACTGGAAGCGTGGGAGCAG
		GCAGGCTGTTCAGCAGCAGAGCTTCGA
	GAPDH	GAGTCTACTGGTGTCTTCACCAC
		CCACAATGCCAAAGTTGTCATGGATGAC
	IL17A	AAAGTCTTTAACTCCCTTGGCGCAAAA
		CTTCCCAGATCACAGAGGGATATC
	SOX2	CCCCAAGATGCACAACTCGGAGATCAGCA
		CCGGTATTTATAATCCGGGTGCTCCT
	STAR	AGAACTTGTGGACCGCATGGAGGCCA
		AATGTGTGGCCATGCCTGCCAGCACA
	SYN1	CGCACACCGACTGGGCAAAATACTTCA
		CCTCACAACTTTGACTCCATTCCG
	TIGAR	CATCATAGACTGTGAGGAAGCACG
		CAAGTCCCTCCCTTCAGAGGCTCCACTGC
For RIP assay	BACE1 3′UTR	TTGCTGATGACATCTCCCTGCTCAAGTA
		GCTCCAACTCATGTGACCAAAGGGA
	BDNF 5′UTR	CTGAGCAAAGCCGAACTTCTCACA
		GCTGTCCTGGAGACTCAGTGTCTTAAAA
	CCND2 5′UTR	TGCCTGAGCGAGAGAGGAGAGCGA
		ACTCGGTCCCGACTGTAAATTTT
	IL17A 3′UTR	CCCTAAGAAACCCCCACGTTTCTC
		CAATTCTGAATCTGCCTCTGAATCCACA
	SOX2 5′UTR	ACAGTCCCGGCCGGGCCGAGGGTTG
		AGCTCCGTCTCCATCATGTTATACA
	STAR 5′UTR	CCACATTGACCTGCAAATCATTGGA
		TATGCAGTGGGAGACACTAGAGA
	SYN1 5′UTR	AGTCTGCAGCGGGCAGCAGAGGAGTC
		AGTTCATGGTGGCGGCGTGGGGCA
	TIGAR 5′UTR	GTAGTGCAGGCAAAGTGGTCCCAGAG
		GCGAAGCGCGGCATTTTGGCACA

### Immunoprecipitation (IP)

The whole cell lysates or nuclear extracts were mixed with 1 μg phosphoserine antibody (ab9332, Abcam) or IgG Rabbit IgG antibody and 40 μl flurry IgA beads (Thermo Fisher Scientific) and rotated overnight at 4°C. Immunoprecipitates were washed by IP buffer (20 mM HEPES [pH 7.9], 350 mM NaCl, 0.1% NP-40, 1 mM DTT, 0.2 mM PMSF, 2 mg/ml leupeptin, and 2 mg/ml aprotinin) and western blotted for the METTL3 antibody.

### Western blot

Brain tissue was homogenized in RIPA buffer solution and then centrifuged at 4°C at 13,000 rpm for 10 min. The protein quantity in the supernatant was determined using a BCA protein assay kit (Well-bio, China). Equal amounts of protein samples were separated by sodium dodecyl sulfate-polyacrylamide gel electrophoresis (SDS-PAGE) and transferred to polyvinylidene fluoride membranes. The membranes were then blocked in 5% nonfat milk and TBS for 90 min and incubated with the respective primary antibodies of METTL3 (1:2000), FTO (1:2000), ALKBH5 (1:2000), IGF2BP1 (1:2000), METTL14 (1:2000, 51104, CST), WTAP (1:2000, 56501, CST), YTHDF1 (1:2000, 17479-1-AP, Proteintech), HNRNPA2B1 (1:2000, 9304, CST), ERK1/2 (1:2000, 67170-1-Ig, Proteintech), p-ERK1/2 (1:500, 80031-1-RR, Proteintech), and GAPDH (1:5000, Beyotime Biotechnology, China) overnight at 4°C. Membranes were washed in TBST and incubated with rabbit anti-mouse and goat anti-rabbit IgG-HRP (1:10000 dilution; 20 ng/ml, Beyotime Biotechnology, China) at room temperature (RT) for 1 h. Membranes were then treated with an enhanced chemiluminescence detection kit (Millipore), and the intensity of each band was quantified by densitometry.

### Statistical analysis

All experimental data were processed and analyzed using SPSS 22.0 statistical software (IBM Corp. Armonk, NY, USA). The measurement data were expressed as the mean ± standard deviation. One-way ANOVA was conducted for comparison among multiple groups. Chi-square analysis was conducted to compare POCD occurrence in mice with and without TPA pretreatment. A *p*-value less than 0.05 was considered statistically significant.
